# Neonatal resuscitation in Eastern Africa: health care providers' level of knowledge and its determinants. A systematic review and meta-analysis

**DOI:** 10.1080/16549716.2024.2396636

**Published:** 2024-09-12

**Authors:** Addis Eyeberu, Elias Yadeta, Deribe Bekele Dechasa, Ahmedin Aliyi Usso, Faysal Mohammed, Adera Debella

**Affiliations:** a School of Nursing and Midwifery, College of Health and Medical Sciences, Haramaya University, Harar, Ethiopia; b School of Public Health, College of Health and Medical Sciences, Haramaya University, Harar, Ethiopia

**Keywords:** Birth asphyxia, sub-Saharan Africa, clinical skills, neonatal resuscitation skills, health care workers

## Abstract

**Background:**

Even though effective neonatal resuscitation prevents the consequences of neonatal death related to birth asphyxia, a significant portion of healthcare personnel lacked understanding or performed it inconsistently. It is critical to have a comprehensive study that demonstrates the overall level of knowledge of healthcare providers regarding neonatal resuscitation in Eastern Africa.

**Methods:**

Articles were searched from Science Direct, JBI databases, Web of Sciences, PubMed, and Google Scholar. The primary outcome was the level of knowledge of health care providers regarding neonatal resuscitation. Data were analyzed using Stata version 18 statistical software. The overall estimates with a 95% confidence interval were estimated using a random effect model.

**Results:**

In this meta-analysis study, 7916 healthcare providers were included. The overall level of knowledge on neonatal resuscitation among healthcare providers in Eastern Africa was 59% [95% CI: 48–70]. Trained health care providers (OR = 3.63, 95% CI: 2.26, 5.00), and work experience of 5 years and above (OR = 2.08, 95% CI: 1.00, 3.16) were determinants of the level of knowledge. However, the level of education and availability of equipment were found to be insignificantly associated with the level of knowledge.

**Conclusions:**

The results of this meta-analysis showed that healthcare professionals in Eastern Africa lacked sufficient knowledge about neonatal resuscitation. Having 5 years of work experience and training in neonatal resuscitation was found to be strongly associated with knowledge level. Thus, continuing education, training courses, and frequent updates on neonatal resuscitation protocols are required for healthcare professionals.

## Paper context

**Main findings:**

The overall level of knowledge on neonatal resuscitation among healthcare providers in Eastern Africa was 59% [95% CI: 48–70].

**Added knowledge:**

This study provides cumulative evidence on the level of knowledge of healthcare providers regarding neonatal resuscitation, and there are regional disparities and inconsistent explanations of risk factors.

**Global health impact for policy and action:**

There is insufficient knowledge among healthcare providers on neonatal resuscitation in Eastern Africa, which can contribute to higher rates of neonatal mortality, highlighting the urgent need for targeted policy interventions.

## Background

It is not uncommon for neonates to have insufficient respiratory efforts when they are born. Approximately 10% of babies need extra respiratory support immediately after birth [1]. The primary cause of neonatal death in countries with limited resources is birth asphyxia [2]. One of the most important evidence-based interventions for birth asphyxia is neonatal resuscitation (NR) [3]. However, the difficulty in implementing these interventions is exacerbated by the fact that up to 60% of deliveries take place outside of medical facilities in sub-Saharan African countries like Ethiopia [4,5] and Tanzania [6,7]. Furthermore, despite implementation of intervention to improve the skill and knowledge of health care professionals, there is a gap in the skill and knowledge of healthcare professionals and the standard of care provided within facilities in sub-Saharan African countries [8,9]. Adequate basic neonatal resuscitation knowledge can prevent several intrapartum-related deaths and reduce the associated complications [1,10,11].

Neonatal death is a huge burden worldwide and more than 88% of neonatal deaths occur in developing countries [12,13]. According to the UNICEF 2019 report, over 2.5 million newborns globally pass away before they even reach the end of their first month of life, with approximately 7000 neonatal deaths every day [14].

There are 28 newborn deaths for every 1000 live births in sub-Saharan African countries, which is 10 times higher than the number of neonatal deaths in high-income countries [14]. It is estimated that two-thirds of 2.5 million neonatal deaths occurring globally may be prevented if basic, high-quality care was provided during intra and post-partum periods [15]. However, healthcare providers (HCPs) in Eastern Africa lack appropriate neonatal resuscitation knowledge and skills. The basic neonatal resuscitation skills are positioning and clearing the airway, suctioning, bag and mask ventilation, drying and stimulation, chest compression, and maintaining warm [16–18]. Every Newborn Action Plan and the Sustainable Development Goals intend to reduce Global rates of neonatal mortality from approximately 20 per 1,000 live births in 2015 to 7 per 1,000 live births by 2035 [19]

Most neonatal deaths in Eastern Africa stem from birth asphyxia, a condition preventable through neonatal resuscitation. However, the situation is exacerbated by the insufficient knowledge of neonatal resuscitation among nurses and midwives [11,20–24]. Studies done in sub-Saharan African countries revealed that the level of knowledge and skill of health professionals regarding neonatal resuscitation before intervention was 70% and 51.9%, respectively [25]. A study done in Ethiopia found that only 37.8% of healthcare providers had adequate knowledge [26]. Another study conducted in Ethiopia showed that 11.2% of health professionals had adequate skills in neonatal resuscitation [27] Despite, the implementation of Helping Baby Breath (HBB) training to improve the healthcare providers’ level of knowledge and skills, a study done in Honduras suggest that following initial training, there is a quick loss of neonatal resuscitation skill on bag and mask ventilation by 1 month [28] Similarly, study done in South Sudan found that despite improvements in knowledge and skills after training, there is significant decline in knowledge and skill after 1 year [29]. Furthermore, to avert neonatal death strategies in addition to HBB, such as essential newborn care practices like skin-to-skin contact, and early initiation of breastfeeding should be implemented [30,31]

There is an increased focus on neonatal resuscitation, which is an evidence-based intervention believed to reduce neonatal mortality from birth asphyxia in Eastern African countries [27,30,32]. healthcare workers in Eastern Africa often exhibit varying levels of practical skill in newborn resuscitation. This variance can be influenced by factors such as access to training, resources, and experience levels [24,33]. Nonetheless, no thorough evaluation or meta-analysis has been conducted to show the combined prevalence of healthcare professionals’ knowledge of newborn resuscitation. Thus, this study aimed to evaluate the pooled level of newborn resuscitation knowledge among healthcare workers in Eastern Africa.

## Methods

### Protocol and registration

The systematic review and meta-analysis study was carried out per Preferred Reporting Items for Systematic Reviews and Meta-Analyses (PRISMA) 2020 guidelines [34] (Supplementary file 1).

### Eligibility criteria

The conditions required for study eligibility were derived from the PICOS criteria. The population consisted of health professionals, such as midwives, nurses, obstetricians, residents, and pediatricians; exposures were determinants that were significantly associated with knowledge level; the study’s outcomes were the health professionals’ level of knowledge regarding neonatal resuscitation; the study’s design was observational studies (cross-sectional studies); and the study’s context was Eastern African countries. Manuscripts that have been published in English before 20 October 2023 were also included. This systematic review and meta-analysis did not consider case series/reports, reviews, commentaries, or editorials.

### Information sources

Articles were searched from legitimate databases such as Science Direct, JBI databases, Web of Sciences, CINAHL (EBOSCO), PubMed, and Google Scholar. We also attempted to access the institutional repository websites of various East African universities such as Addis Ababa University, Open University of Tanzania, Kenyatta University, and University of Dodoma for unpublished articles.

### Search strategy and study selection

Boolean logic operators (AND, OR, and NOT), Medical Subject Headings (MeSH), and keywords were combined to perform the search. Sample search strategy used for PubMed was (‘infant, newborn’[MeSH Terms] OR (‘infant’[All Fields] AND ‘newborn’[All Fields]) OR ‘newborn infant’[All Fields] OR ‘neonatal’[All Fields] OR ‘neonate’[All Fields] OR ‘neonates’[All Fields] OR ‘neo natality’[All Fields] OR ‘neonatal’[All Fields] OR ‘neonate s’[All Fields]) AND (‘resuscitability’[All Fields] OR ‘resuscitate’[All Fields] OR ‘resuscitated’[All Fields] OR ‘resuscitates’[All Fields] OR ‘resuscitating’[All Fields] OR ‘resuscitation’[MeSH Terms] OR ‘resuscitation’[All Fields] OR ‘resuscitations’[All Fields] OR ‘resuscitative’[All Fields] OR ‘resuscitator’[All Fields] OR ‘resuscitators’[All Fields]) AND (‘health personnel’[MeSH Terms] OR (‘health’[All Fields] AND ‘personnel’[All Fields]) OR ‘health personnel’[All Fields] OR (‘health’[All Fields] AND ‘care’[All Fields] AND ‘providers’[All Fields]) OR ‘health care providers’[All Fields]) AND (‘Africa, eastern’[MeSH Terms] OR (‘Africa’[All Fields] AND ‘eastern’[All Fields]) OR ‘eastern Africa’[All Fields] OR (‘eastern’[All Fields] AND ‘Africa’[All Fields])). Furthermore, the name of each country in eastern Africa is used in Google search. The countries listed in the google are Burundi, Comoros, Djibouti, Ethiopia, Eritrea, Kenya, Rwanda, Seychelles, Somalia, South Sudan, Sudan, Tanzania, and Uganda.

The articles were exported to reference management software (Endnote™ version X8) following a search of articles from websites and databases. After that, duplicate articles were removed manually. Subsequently, the titles and abstracts of the remaining publications were screened. Articles that did not meet the eligibility requirements were removed. The full texts of the remaining publications were then independently reviewed by two authors (AD and AA). We next carried out a more comprehensive analysis of the study objective, inclusion and exclusion criteria, study population, measurement, sample size, and main findings. The two authors (DB and EY) came to a rational consensus over how to address any queries that surfaced during the extraction process with each other’s assistance.

### Data extraction and data item

The data were extracted by two authors (EY and FM) separately. Variables considered for extraction were author and year, country, data collection method, study design, the primary outcome of interest, knowledge level, and significant variables (Adjusted odds ratios (AOR) with their 95% CI). The results generated by the two authors (AE and DB) were compared to verify the accuracy of the data extraction. The variables author and year, sample size, event, odds ratio, lower confidence interval, and higher confidence were extracted for the meta-analysis of primary and secondary outcomes.

The primary outcome of this study was the level of knowledge of healthcare providers regarding neonatal resuscitation in Eastern Africa. The secondary outcome of interest was determinants of the level of knowledge of health care providers regarding neonatal resuscitation in Eastern Africa.

### Quality of study

The studies’ quality was evaluated using the cross-sectional Joana Briggs Institute (JBI) criteria [35]. The listed studies’ methodological validity and reliability were assessed. Two authors (EY and BB) assessed and scored the study’s quality using JBI. The eight JBI criteria were used to classify the collected research into three quality categories: high, moderate, and low. A score of 60% or less is deemed low quality, 60% to 80% is considered moderate quality, and 80% or more is considered good quality.

### Statistical analysis

The statistical analysis was performed using the statistical software Stata version 18 [36]. The overall estimate of healthcare professionals’ level of knowledge about newborn resuscitation and its determinants in Eastern Africa was presented using forest plots. A meta-analysis was carried out using a random effect model to lessen the heterogeneity of the included studies. Furthermore, subgroup analysis, sensitivity analysis, and bivariate and multivariate meta-regression were done. Leave-one-out meta-analysis method was used for sensitivity analysis to determine the impact of a single study on the total estimate and to identify any outliers. Based on the I2 statistic recommendations provided by Higgins et al. (an I2 of 75/100% and above implying considerable heterogeneity), a meta-analysis of observational studies was conducted. The researchers used visual evaluation of a funnel plot, trim fill analysis, and Egger’s Regression Test to search for possible publication bias.

## Results

### Search finding and risk of bias assessment

**Figure 1.**
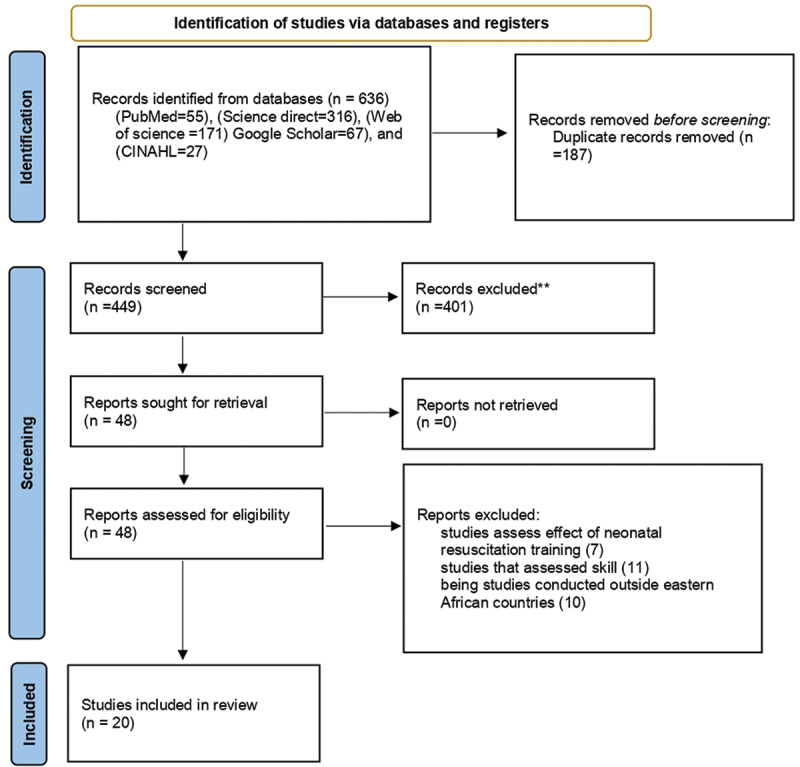
PRISMA 2020 flow diagram for systematic reviews and meta-analysis of the level of knowledge of health care providers regarding neonatal resuscitation in Eastern Africa, 2023.

A total of 636 × published articles were found on reputable databases and websites. A total of 187 publications were removed by EndNote™ and visual inspection due to duplication. After that, the titles and abstracts of the 449 studies were retained and screened. After being scrutinized based on abstracts and titles, 401 in total were eliminated. Of the 48 publications that were deemed appropriate, 28 were excluded due to their evaluation of the impact of newborn resuscitation training, their skill assessment component, and the fact that they were carried out outside of nations in East Africa. At last, our study contained 20 papers that satisfied the qualifying requirements (Figure 1).

All studies received a score ranging from 5 to 7, with the majority of the studies receiving a 7. Fourteen studies received seven out of eight possible points, three studies received six out of eight possible points, and three studies received five out of eight possible points. The studies were deemed to be eligible to be included in the analyses (Supplemental file 2).

### Characteristics of included studies

This systematic review and meta-analysis included 20 observational studies (19 cross-sectional and one mixed studies). Of the total studies, 11 studies were conducted in Ethiopia [26,33,37–45], 3 studies were conducted in Tanzania [46–48], 3 studies were conducted in Kenya [49–51], 2 studies were conducted in Uganda [52,53], and 1 study conducted in Somalia [54]. All of the included studies assessed both prevalence and factors associated with healthcare providers’ level of knowledge on neonatal resuscitation in Eastern Africa. The sample size of the included studies ranges from 30 in a study conducted in Uganda [53] to 3804 in a study conducted in Ethiopia [37]. Of the included studies, 16 (80%) of the studies were published after 2020. In terms of data collection methods, five of the included studies used face-to-face interviews to collect data, six studies used self-administered interviews and direct observation to collect data, four studies used self-administered interviews to collect data, four studies used face-to-face interviews and direct observation to collect data, and one study collected data using chart review. In all, 7916 healthcare providers were included in this systematic review and meta-analysis to examine the HCP’s level of knowledge on neonatal resuscitation in Eastern Africa. Table 1 presents a summary of the key features of the papers that were part of this systematic review and meta-analysis (Table 1).

**Table 1.** Characteristics of included studies in this systematic review and meta-analysis in 2023.

Author year	Country	Design	Data collection method	Participants	Sample size	Prevalence	Significant variables with 95 CI
Sintayehu Y et al., 2020 [40]	Ethiopia	CS	Interview	MW&N	427	9.8	Being trained on newborn resuscitation (AOR = 3.79, 95% CI: 1.73, 8.32), being unmarried (AOR = 2.36, 95% CI: 1.11, 5.02), holding bachelor sciences degree or above (AOR = 2.67, 95% CI: 1.11, 6.47), and working under west Hararge health institutions (AOR = 0.30, 95% CI: 0.10, 0.88).
Gebreegziabher et al., 2014 [33]	Ethiopia	CS	Self-administered questionnaire	HCP	135	43	
Abrha et al., 2019, [37]	Ethiopia	CS	Review	HCP	3804	49	Trained on neonatal resuscitation (*β* = 2.65, 95% CI: 0.65, 4.62; *p* < 0.00), facilities that had guideline of neonatal resuscitation (*β* = 2.50, 95% CI: 0.60, 3.52; *p* = 0.01), and availability of essential equipment (*β* = 0.95, 95% CI: 0.44, 1.45; *p* = 0.02)
Biset, G et al., 2023 [26]	Ethiopia	CS	Interview	MW&N	143	37.8	On job training [*p* < 0.01], presence of Neonatal Resuscitation guide in the working unit [*p* < 0.02], got supportive supervision within preceding six months of this study [*p* < 0.05]
Bekele, FA et al., 2021 [11]	Ethiopia	CS	Interview	MW&N	306	46.5	Sex (AOR = 2.33, 95% CI (1.38–3.95)), participants in neonatal resuscitation training (AOR = 7.81, 95% CI (4.37–13.96)), and the field of study or profession (AOR = 0.142, 95% CI (0.63–0.32)) were the significant factors associated with the knowledge of nurses and midwives of neonatal resuscitation.
Mersha A et al., 2020 [22]	Ethiopia	CS	Interview and observation	HCP	429	76.2	Age, training, recent involvement in basic newborn resuscitation, and the well-equipped facility
Bogale M et al., 2021 [42]	Ethiopia	CS	self-administered questionnaires and observation	MW&N	172	89	Age, training, recent involvement in basic newborn resuscitation, and the well-equipped facility
Wayessa, ZJ et al., 2021 [43]	Ethiopia	CS	Interview	HCP	402	79.4	
Fekede, Ayantu, 2023	Ethiopia	CS	Interview	MW&N	163	66.3	Training were 2.6 times {AOR = 2.6; 95% CI (1.02- 6.63)},
Mbinda, MA, 2021 [46]	Tanzania	CS	self-administered questionnaires and observation	MW&N	340	94	BSC degree (AOR = 5.51, *p* < 0.03) compared to certificates
Abebaw, M et al., 2022 [45]	Ethiopia	CS	self-administered questionnaires	HCP	409	87.3	
Ahmed MA, 2022 [54]	Somalia	CS	Interview and observation	MW&N	40	15	
Joho AA, et al., 2020 [21]	Tanzania	CS	self-administered questionnaires and observation	HCP	172	41.1	Experience in the maternity unit (AOR = 2.94; CI: 0.96–8.98)
Kamau PT, et al., 2022 [49]	Kenya	CS	self-administered questionnaires and observation	HCP	46	46	
Muli DM, 2021 [50]	Kenya	CS	Interview and observation	N	201	56	
Murila F, et al., 2012 [51]	Kenya	CS	self-administered questionnaires	HCP	192	35.4	
Mzurikwao CB, et al., 2018 [48]	Tanzania	CS	self-administered questionnaires	HCP	330	42.4	Working on hospitals (AOR = 3.227, 1.992-5.229), Nurse (AOR-3.118, 1.62-5.999), training (AOR = 1.778, 1.003, 3.154)
Shinde S, et al., 2022 [44]	Ethiopia	Mixed	Interview and observation	HCP	100	54	
Namuguzi M, et al., 2020 [52]	Uganda	CS	self-administered questionnaires and observation	MW&N	75	92	
Kembabazi R 2023 [53]	Uganda	CS	self-administered questionnaires and observation	MW	30	99	

Note: CS: Cesarean section, HCP: Health Care Providers, MW: Midwives, N: Nurses, MW&N: Midwives and Nurses.

### Meta-analysis of the primary outcome

**Figure 2.**
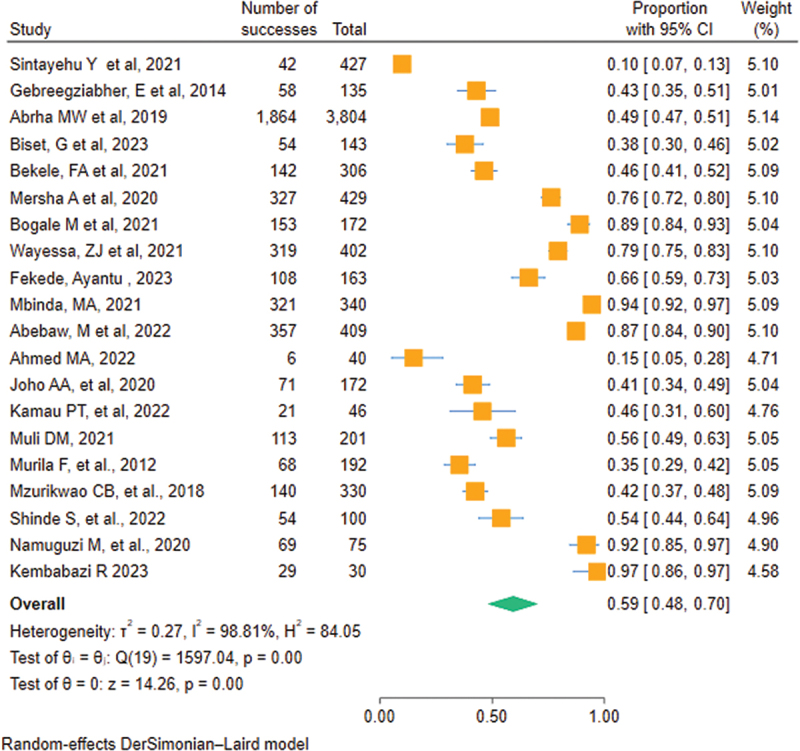
Forest plot on the pooled level of knowledge of health care providers regarding neonatal resuscitation in Eastern Africa, 2023.

A total of 20 studies were included in a meta-analysis of the healthcare providers’ level of knowledge on neonatal resuscitation in Eastern Africa. The level of knowledge on neonatal resuscitation in Eastern Africa ranges from 10% (95% CI: 7–13) in a study done in Ethiopia to a maximum of 97% (95% CI: 86–97) in a study done in Uganda. The pooled level of knowledge on neonatal resuscitation among healthcare providers in Eastern Africa was 59% [95% CI: 48–70]. There is a significant heterogeneity among individual studies (*i*^2^ = 98.81%), Cochrane Q statistics is significant (*p* = ≤0.0001), and the confidence interval of some of the estimates in the forest plot does not overlap (Figure 2).

### Subgroup analysis of the level of knowledge of HCP on neonatal resuscitation

**Figure 3.**
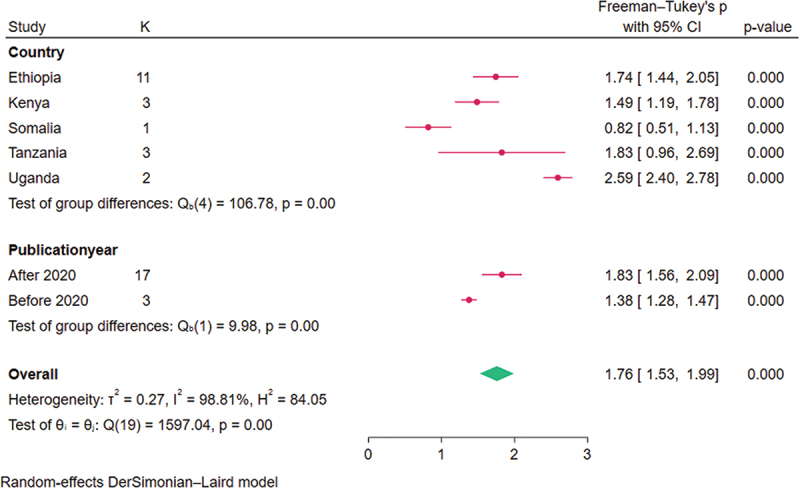
Forest plot on subgroup analysis of the level of knowledge of health care providers regarding neonatal resuscitation in Eastern Africa, 2023.

Based on subgroup analysis by country, the highest level of knowledge on neonatal resuscitation among healthcare providers in Eastern Africa was observed among studies conducted in Uganda 94% (95% CI: 88–98). The lowest level of knowledge on neonatal resuscitation among health care providers in Eastern Africa was observed among studies conducted in Somalia 15% (95% CI: 5–28). Furthermore, the highest heterogeneity between studies (*i*^2^ = 99.37%) was observed among studies conducted in Uganda, while the lowest heterogeneity between studies (*i*^2^ = 0.00%) was observed in studies conducted in Tanzania (Figure 3).

Based on subgroup analysis by the publication year, the highest level of knowledge on neonatal resuscitation among healthcare providers in Eastern Africa was observed among studies conducted after 2020 which was 63% (95% CI: 50–75) (Figure 3).

### Heterogeneity assessment

Heterogeneity was evaluated using both graphical and statistical methods. Concerning statistical methods, the overall level of knowledge of neonatal resuscitation among health care professionals in Eastern Africa indicated significant heterogeneity, as evidenced by significant results from the Cochrane Q statistics (*p* ≤ 0.001), test of theta (*p* ≤ 0.001), and I-square test statistics (I2 = 98.81). Plotting-wise, the Galbraith plot demonstrated that a single estimate was outside of the 95% confidence interval, indicating a notable degree of study heterogeneity (Supplementary file 3).

### Multivariate meta regression

To ascertain the reason for heterogeneity, a meta-regression analysis was carried out, taking into consideration variables such as countries, sample size, and publication year. It was discovered that none of the variables were the cause of the heterogeneity. Thus, variability may result from additional study-level characteristics that were not considered in this analysis (Table 2).

**Table 2.** Multi-variate meta-regression analysis of the level of knowledge of health care providers regarding neonatal resuscitation in Eastern Africa.

Variables	Coefficients	Standard error	*p*	95% CI
Sample size	5.0406	0.000185	0.978	−0.0003575, 0.0003676
Country	0.0747944	0.1027672	0.467	−0.1266257, 0.2762145
Year of publication	0.055768	0.0519371	0.283	−0.0460268, 0.1575628

### Sensitivity analysis

To evaluate the impact of a single study on the overall estimate of the remaining studies in the meta-analysis results and to find outliers, a leave-one-out meta-analysis was employed. There was no discernible difference between the initial estimate and the revised estimate of the pooled level of newborn resuscitation knowledge among healthcare workers in Eastern Africa after individual studies were eliminated (Supplementary file 4). Conversely, the confidence intervals for individual research overlap.

### Publication bias

**Figure 4.**
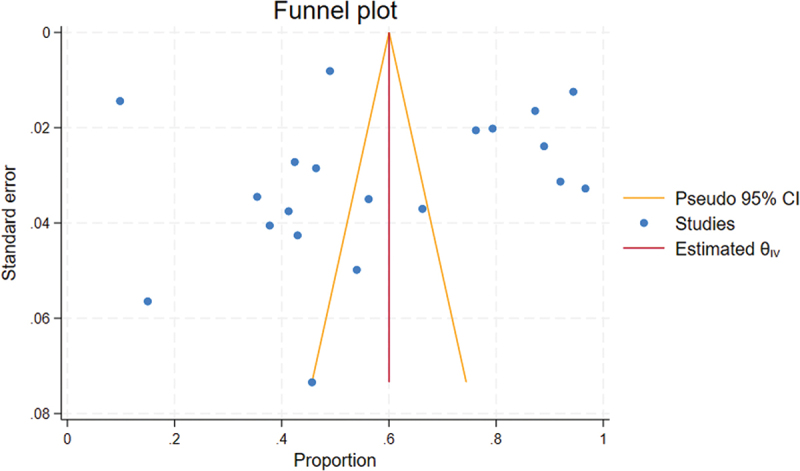
Funnel plot of level of knowledge of health care providers regarding neonatal resuscitation in Eastern Africa, 2023.

The funnel plot appears asymmetrical upon visual inspection, which may be related to significant heterogeneity between the included studies (Figure 4). Egger’s test indicated that there was no small study impact (*p* = 0.192). Moreover, there was no difference between the observed and the combination of observed and imputed effect size estimates in the random effect model, as per the nonparametric trim-and-fill study of publication bias.

## Meta-analysis of the factors associated with knowledge of health care providers on neonatal resuscitation

### The association between having training on neonatal resuscitation and knowledge

**Figure 5.**
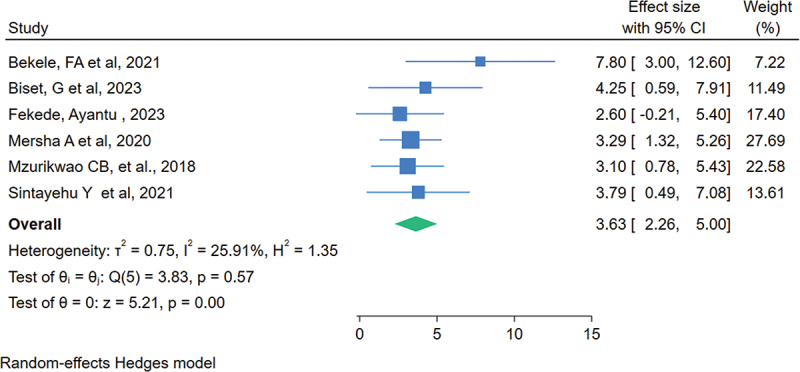
The association between training and level of knowledge of health care providers regarding neonatal resuscitation in Eastern Africa, 2023.

Six studies out of the 20 examined the relationship between healthcare providers’ level of knowledge regarding neonatal resuscitation and their training in neonatal resuscitation. The overall estimated effect size demonstrated that training is associated with a higher level of neonatal resuscitation knowledge among healthcare providers in Eastern Africa. Healthcare professionals who received neonatal resuscitation training were 3.63 times more likely to be knowledgeable about neonatal resuscitation than those who did not (OR = 3.63, 95% CI: 2.26, 5.00) (Figure 5).

### The association between having work experience of 5 and above years and knowledge

Of the total included studies, three studies examined the association between having work experience of 5 and above years and knowledge of HCP on neonatal resuscitation in Eastern Africa.

**Figure 6.**
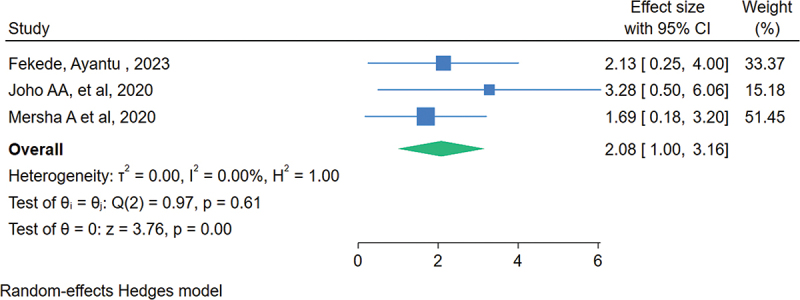
The association between work experience and level of knowledge of health care providers regarding neonatal resuscitation in Eastern Africa, 2023.

The overall estimate showed that there was a statistically significant association between having work experience of 5 and above years and knowledge among healthcare providers in Eastern Africa. The odds of having a good level of knowledge were 2.08 times higher among health care providers who had work experience of 5 and above than health care providers who had work experience of less than 5 (OR = 2.08, 95% CI: 1.00, 3.16) (Figure 6).

### The association between level of education and knowledge

Of the total included studies, three studies examined the association between the level of education and knowledge among healthcare providers in Eastern Africa. The overall estimate showed that there was no statistically significant association between the level of education and knowledge of health care providers (OR = 1.04, 95% CI: 0.15, 2.23) (Supplementary file 5).

### The association between the availability of equipment and knowledge

Of the total studies, three studies assessed the association between the availability of equipment and knowledge among healthcare providers in Eastern Africa. The overall estimate showed that there was no statistically significant association between the availability of equipment and knowledge among healthcare providers in Eastern Africa. (OR = 1.42, 95% CI: 0.40, 2.44) (Supplementary file 6).

## Discussion

Low-income countries continue to face a serious public health concern with newborn mortality. The best way to lessen the burden of newborn mortality is through interventions like neonatal resuscitation. Nonetheless, there is little information available about the level of newborn resuscitation knowledge among healthcare providers in Eastern Africa. Therefore, this systematic review and meta-analysis aimed to ascertain the level of knowledge of healthcare providers regarding newborn resuscitation in Eastern Africa. According to the results of this meta-analysis, the overall level of knowledge on neonatal resuscitation among healthcare providers in Eastern Africa was 59% [95% CI: 48–70]. Trained health care providers (OR = 3.63, 95% CI: 2.26, 5.00), and work experience of 5 years and above (OR = 2.08, 95% CI: 1.00, 3.16) were determinants of the level of knowledge.

According to the findings of this meta-analysis, the pooled level of knowledge on neonatal resuscitation among healthcare providers in Eastern Africa was 59% [95% CI: 48–70]. This finding is similar to studies conducted in low-income countries such as Ghana [55] and Nigeria [56]. Moreover, the finding is lower than studies done in Afghanistan [57] and Nigeria [58] (78%). The findings of this meta-analysis clearly show that many healthcare providers have not acquired the necessary knowledge regarding neonatal resuscitation in Eastern Africa (41%). This may be due to a lack of short-term and long-term training specific to neonatal health, a lack of manuals and guidelines, and refresher training. According study done in Uganda highlighted that clinical practice alone is not enough to retain knowledge and refresher training is required [59]. Furthermore, the findings of this study demonstrated that the healthcare providers’ level of knowledge is not at the expected level and requires the intersectoral collaborations of the Ministry of Health of each country and non-governmental organizations to increase the level of knowledge. To improve the knowledge of East African healthcare providers, frequent and intensive courses on neonatal resuscitation are required [60].

In this study, the overall summary estimate showed that having training is significantly associated with healthcare providers’ adequate level of knowledge. Healthcare providers who took training on neonatal resuscitation were 3.63 times more likely to be knowledgeable on neonatal resuscitation than those who did not take training. This result is supported by a systematic review and meta-analysis study which illustrated that the majority of healthcare providers who took training were more likely to acquire and retain knowledge regarding neonatal resuscitation. Furthermore, the evidence outlined that refresher training seemed to improve retention and then reduce newborn mortality in low-income countries [24]. Findings from Nigeria showed that immediately after training, the level of knowledge increased [56]. These findings implied that training is an important issue in updating the health care providers regarding neonatal resuscitation and needs to be given to all health care providers who work in neonatal intensive care units and labor and delivery units. After 2010, Helping Baby Breath (HBB) training was given to healthcare providers in low- and middle-income countries and found to improve newborn survival by reducing neonatal death from birth asphyxia [61]. Other evidence also showed that neonatal resuscitation training reduces neonatal mortality [62,63]. Thus, it is imperative to strengthen and continue the provision of neonatal resuscitation training. However, despite the importance of training and knowledge, there are instances where improvements in clinical practice and neonatal survival after resuscitation do not always occur. Evidence showed that training improves HCP simulation performance but not clinical management of neonates [64]. To guarantee continuous gains in clinical practice and newborn outcomes following resuscitation, supportive settings, and continuous quality improvement initiatives must be combined with training and knowledge, which are crucial [65].

In this study, the odds of having an adequate level of knowledge were higher among healthcare providers who had work experience of 5 years than their counterparts. This finding is supported by studies conducted in Nigeria [58]. This may be due to healthcare providers working in the health facility for more than 5 years who may get a chance for neonatal resuscitation training and ongoing educational opportunities. As the level of knowledge increases, the ability to conduct neonatal resuscitation will increase and then improve neonatal survival.

## Strengths and limitations of the study

One of the study’s strongest points is that the majority of the publications were found in recent years and were obtained from a range of reliable databases. To the best of the researchers’ knowledge, there is a lack of comprehensive data that shows the level of knowledge of healthcare providers regarding neonatal resuscitation in Eastern Africa. However, most of the pieces originated in a few countries. Only the English language was considered when searching for articles, which could have an impact on the overall estimate.

## Implication of finding

Inadequate level of knowledge of health care providers regarding neonatal resuscitation has serious implications for newborn health. It may lead to a delay in initiating or performing neonatal resuscitation, which increases the risk of adverse neonatal outcomes. Inadequate resuscitation due to lack of knowledge may contribute to a higher risk of neonatal mortality, long-term health consequences such as neurological damage and developmental delay, and poor quality of life. Furthermore, healthcare providers may face legal and ethical challenges for adverse neonatal outcomes. Therefore, ongoing education, training programs, and regular updates on neonatal resuscitation guidelines for healthcare providers are necessary. Continual improvement in knowledge level can enhance the quality of neonatal resuscitation, ultimately improving the survival of newborns.

## Conclusion

The finding of this meta-analysis revealed that the level of knowledge of healthcare providers regarding neonatal resuscitation in Eastern Africa was not adequate. Trained health care providers and work experience of 5 years and above were significantly associated with level of knowledge. Thus, both the government and all the concerned stakeholders should take coordinated action to facilitate short-term and refresher training to improve knowledge levels.

## Supplementary Material

Supplemetary file 5.tif

Supplementary file 4.tif

Supplementary file 1.docx

Supplemetary file 6.tif

Supplementary file 2.docx

Supplementary file 3.tif
