# Supplementary figures and images for: Neonatal resuscitation in Eastern Africa: health care providers' level of knowledge and its determinants. A systematic review and meta-analysis
Source: Glob Health Action. 2024 Sep 12;17(1):2396636. doi: 10.1080/16549716.2024.2396636 (PMC11395871; doi:10.1080/16549716.2024.2396636)

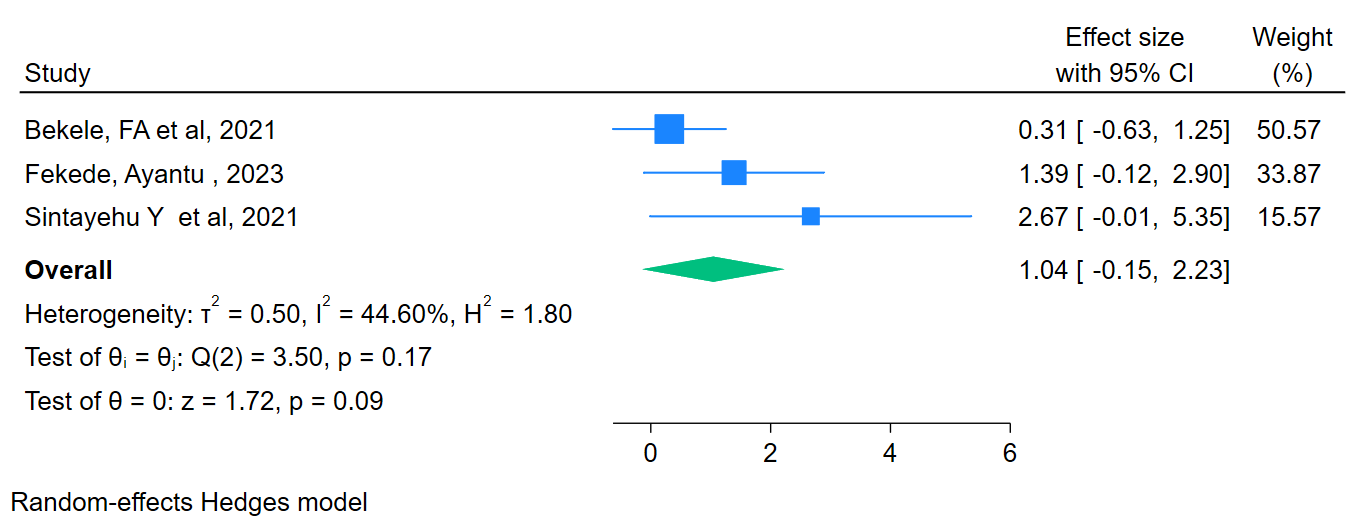

Supplement: Supplemetary file 5.tif [file ZGHA_A_2396636_SM2065.tif]

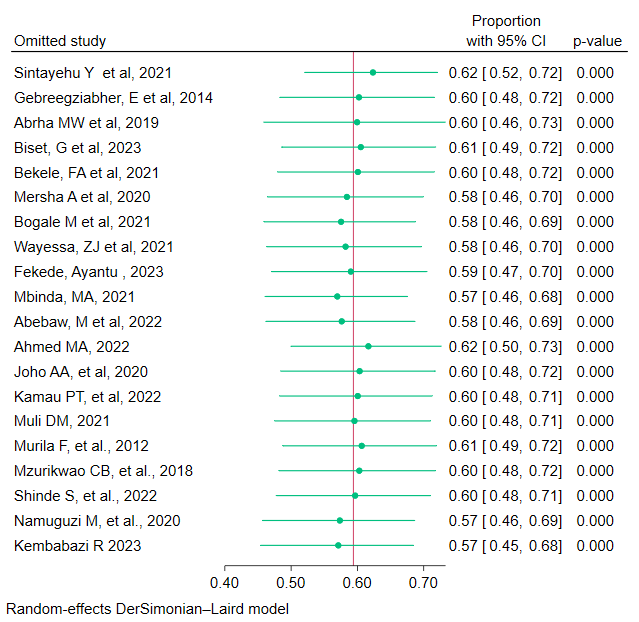

Supplement: Supplementary file 4.tif [file ZGHA_A_2396636_SM2064.tif]

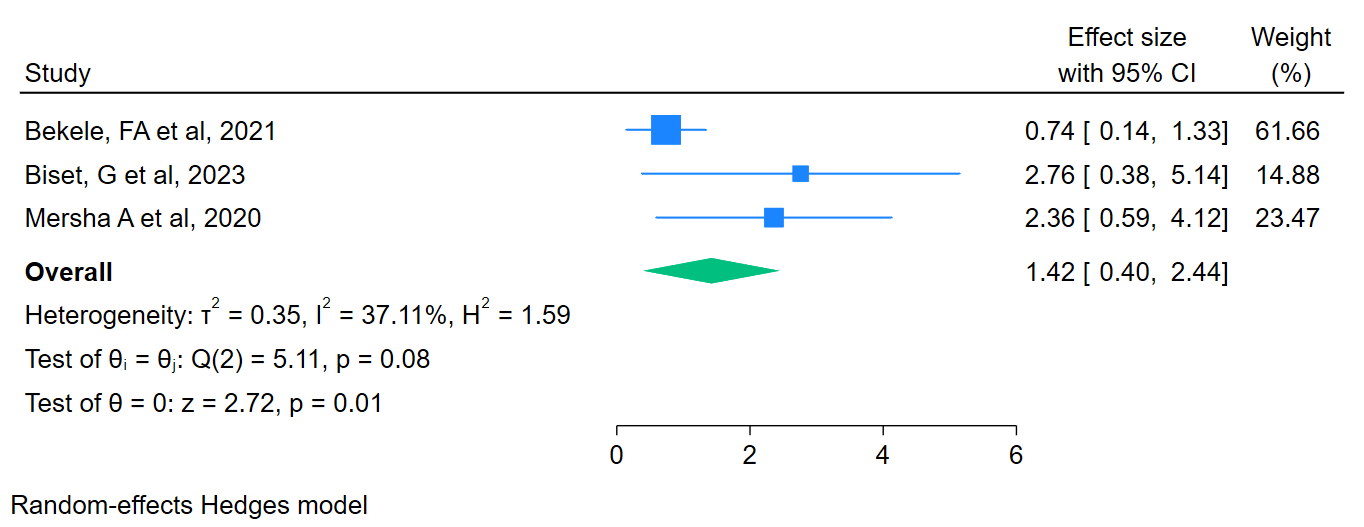

Supplement: Supplemetary file 6.tif [file ZGHA_A_2396636_SM2062.tif]

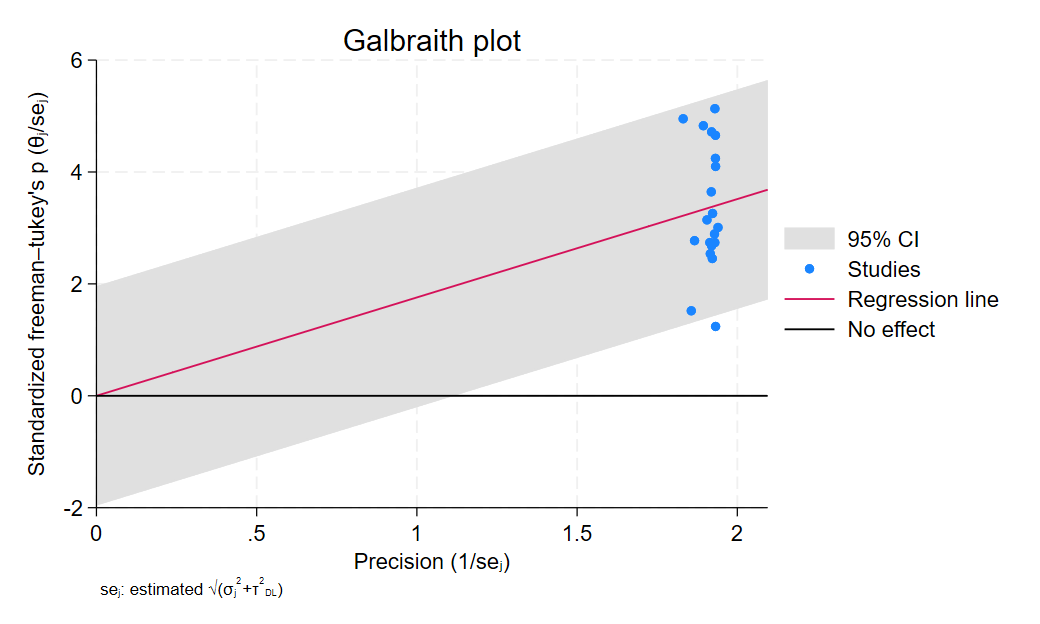

Supplement: Supplementary file 3.tif [file ZGHA_A_2396636_SM2060.tif]
