# Supplementary material for: Neonatal resuscitation in Eastern Africa: health care providers' level of knowledge and its determinants. A systematic review and meta-analysis
Source: Glob Health Action. 2024 Sep 12;17(1):2396636. doi: 10.1080/16549716.2024.2396636 (PMC11395871; doi:10.1080/16549716.2024.2396636)
Supplement: Supplementary file 2.docx [file ZGHA_A_2396636_SM2061.docx]

**Appraisal**

Table 1: Critical appraisal check list for the assessment of overweight and obesity and its determinant among adolescents in Ethiopia (1 =yes, 0=no/not mentioned); total score=8

| **Studies** | Q1 | Q2 | Q3 | Q4 | Q5 | Q6 | Q7 | Q8 | Total score | Remark |
| --- | --- | --- | --- | --- | --- | --- | --- | --- | --- | --- |
| Sintayehu Y et al, 2021 | Y | Y | Y | Y | N | Y | Y | Y | 7/8 |  |
| Gebreegziabher, E et al, 2014 | Y | Y | Y | Y | N | Y | Y | Y | 7/8 |  |
| Abrha MW et al, 2019 | Y | Y | Y | Y | N | Y | Y | Y | 7/8 |  |
| Biset, G et al, 2023 | N | Y | Y | Y | N | Y | Y | Y | 7/8 |  |
| Bekele, FA et al, 2021 | N | Y | Y | Y | N | Y | Y | Y | 7/8 |  |
| Mersha A et al, 2020 | Y | Y | Y | Y | U | Y | Y | Y | 7/8 |  |
| Bogale M et al, 2021 | N | Y | Y | Y | N | Y | Y | Y | 7/8 |  |
| Wayessa, ZJ et al, 2021 | Y | Y | Y | Y | N | Y | Y | Y | 7/8 |  |
| Fekede, Ayantu , 2023 | N | Y | Y | Y | N | Y | Y | Y | 6/8 |  |
| Mbinda, MA, 2021 | Y | Y | Y | Y | N | Y | Y | Y | 7/8 |  |
| Abebaw, M et al, 2022 | N | N | Y | Y | N | Y | Y | Y | 6/8 |  |
| Ahmed MA, 2022 | N | Y | Y | Y | N | Y | Y | Y | 6/8 |  |
| Joho AA, et al, 2020 | Y | Y | Y | Y | N | Y | Y | Y | 7/8 |  |
| Kamau PT, et al, 2022 | N | N | Y | Y | N | Y | Y | Y | 5/8 |  |
| Muli DM, 2021 | Y | Y | Y | Y | N | Y | Y | Y | 7/8 |  |
| Murila F, et al., 2012 | Y | Y | Y | Y | N | Y | Y | Y | 7/8 |  |
| Mzurikwao CB, et al., 2018 | Y | Y | Y | Y | N | Y | Y | Y | 7/8 |  |
| Shinde S, et al., 2022 | Y | Y | Y | Y | N | Y | Y | Y | 7/8 |  |
| Namuguzi M, et al., 2020 | N | Y | Y | Y | N | Y | Y | Y | 5/8 |  |
| Kembabazi R 2023 | N | Y | Y | Y | N | Y | Y | Y | 5/8 |  |

Notes:

Q1 - Were the criteria for inclusion in the sample clearly defined?

Q2 - Were the study subjects and the setting described in detail?

Q3 - Was the exposure measured in a valid and reliable way?

Q4 - Were objective, standard criteria used for measurement of the condition?

Q5 - Were confounding factors identified?

Q6 - Were strategies to deal with confounding factors stated?

Q7 - Were the outcomes measured in a valid and reliable way?

Q8 - Was appropriate statistical analysis used?

Abbreviations: Y, yes; N, no; U, unclear.
